# Domestic Summary

**Published:** 1839-09-21

**Authors:** 


### DOMESTIC SUMMARY.

*Louisville Medical Institute.*—The annual circular, for 1839, of this institution, presents a list of 120 students, and 27 graduates. We perceive that Professor Drake, late of Cincinnati, has been appointed to the chair of Clinical Medicine and Pathological Anatomy. In announcing this appointment, the Board of Managers very justly observe, that Dr. Drake's mature age,—his long experience in the practice of his profession,—his great powers and popularity as a teacher,—the high estimate in which he is held by the profession throughout the Union, as manifested by his successive appointments to different chairs in the Medical College of Ohio, the Miami University, the Cincinnati College, Transylvania University, and Jefferson Medical College of Philadelphia, are so many warrants of the propriety of the measure just taken, and pledges of the benefits to be expected from it, in the great and important cause of medical education.

---

*Amputation at the Shoulder-Joint.*—On Monday, the 2d September, Dr. Walker, of Charlestown, amputated at the shoulder-joint on a lad at Lynn, under the following very peculiar circumstances. On the 19th of August, A. E. Blood, aged thirteen years, was kicked on the shoulder by a horse, while his hand was on his head. The axilla filled immediately. A physician was sent for, who, finding the parts much swollen, but no evidence of any thing but a contusion, prescribed the remedies usual in such cases. Eight days from the accident there was a protrusion in the axilla, like a pullet's egg, of a bluish colour. By the advice of Professor G——, of N. Y. University, on the fourteenth day, an incision was made through the integuments just over the inferior edge of the pectoralis major. No bleeding or matter flowed from the incision; there was a little bloody serum. The operator not caring to look deeper for *matter*, merely dressed the wound lightly. The next day arterial hæmorrhage occurred from the wound. This was arrested by a compress and bandage, but not so but that it followed again the next day and the day after. It was at this juncture that Dr. Walker was sent for. He found the lad pallid from loss of blood, having lost, as was estimated by the physician, full two quarts. The parts about the shoulder were very much swollen; there was no pulsation at the wrist, or in the axilla. On examination, pulsation was perceived in the tumour. The arm was of nearly natural colour and temperature, with some appearance of commencing œdema.

Dr. Walker stated to the friends the nature of the case, for the cure of which he recommended one of two operations; either the tying of the subclavian, or amputation at the shoulder-joint. He stated the difficulties and dangers of both operations—the comparative safety of the latter, and the inevitably fatal result of the former, if, in cutting down for the artery, he should cut into the aneurismal cavity, and should be unable to find readily and secure the mouth of the bleeding artery. On a view of all the circumstances of the case, the friends desired Dr. Walker to amputate, which he proceeded to do, the subclavian being compressed, as it passes out of the chest, by an assistant. The arteries were secured by ligature, as they were cut, to prevent further loss of blood. On removing the coagula, about sixteen ounces, after the amputation, and looking for the mouth of the artery from which the bleeding came, the subclavian was found completely severed transversely as it passes under the clavicle, the separated ends of which were an inch and a half apart—so that had an attempt been made to tie the subclavian, the lad could hardly have escaped with his life. In what way was the wound of the artery produced? There was no external wound, and the artery was cut short off as clean as though it had been done with a knife. The lad is now, Sept. 9th, doing well.—*Boston Med. and Surg. Journal.*

